# Supplementary material for: Drug Response of Patient-Derived Lung Cancer Cells Predicts Clinical Outcomes of Targeted Therapy
Source: Cancers (Basel). 2024 Feb 14;16(4):778. doi: 10.3390/cancers16040778 (PMC10887363; doi:10.3390/cancers16040778)
Supplement: Supplementary file 1 [file cancers-16-00778-s001.zip › 4_PDC Supple Figures_231226.pdf]

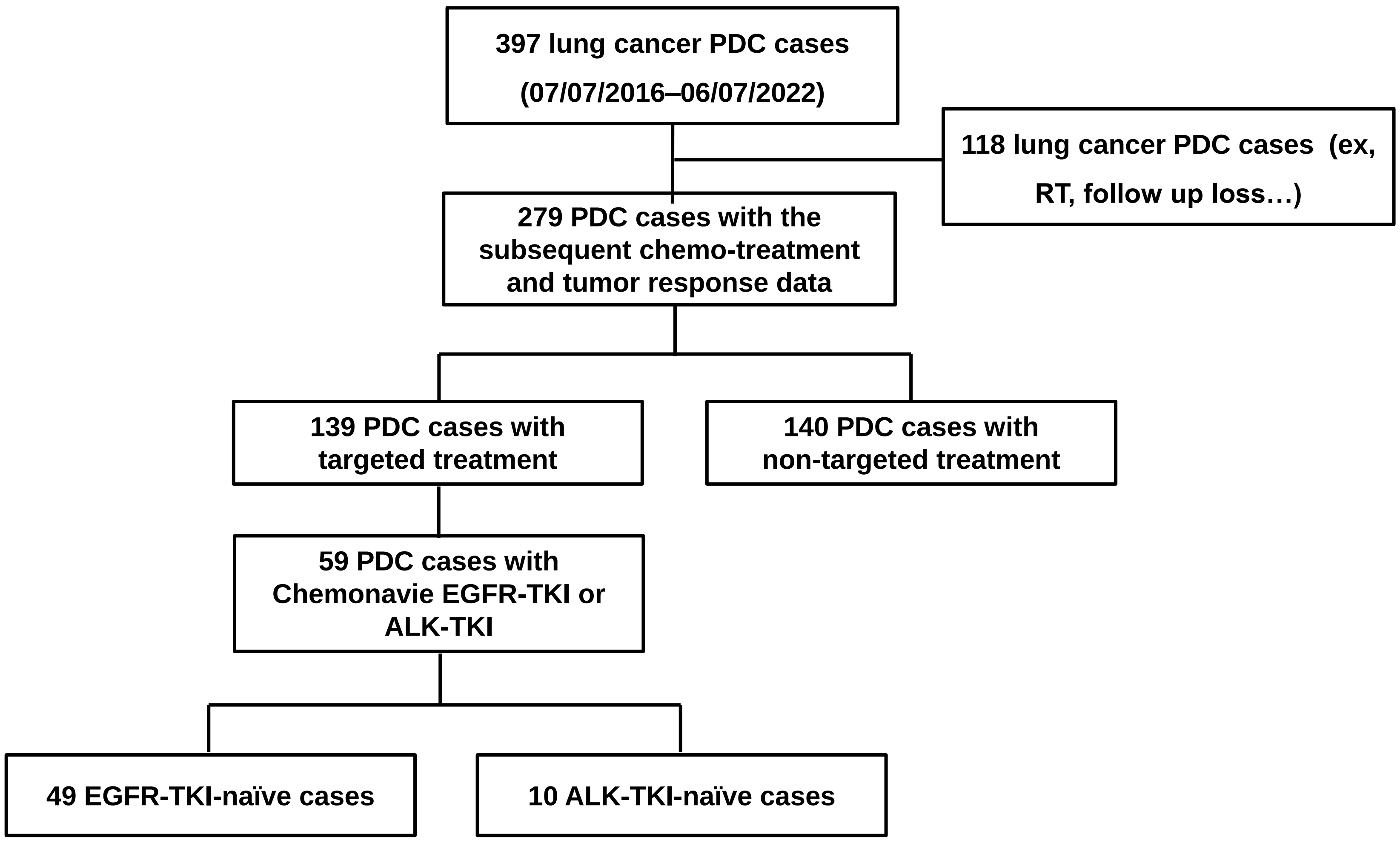

Figure S1. Consort diagram based on the cases of 59 chemo-naive *EGFR*- or *ALK*-positive NSCLC patient-derived cell cultures (PDC). Abbreviations: EGFR-TKI, EGFR tyrosine kinase inhibitor; ALK-TKI, ALK-tyrosine kinase inhibitor.

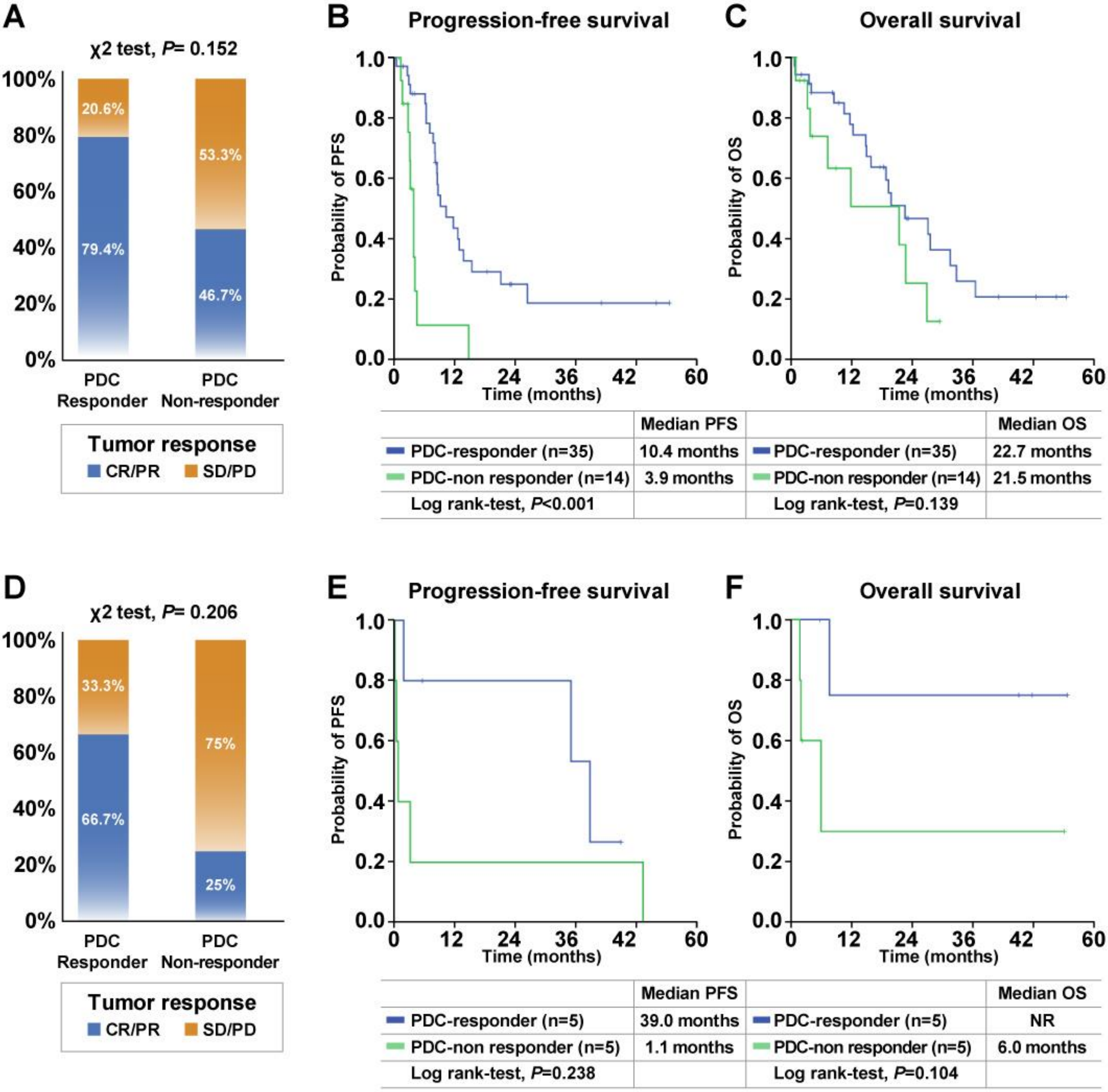

Figure S2. Associations of PDC drug response with clinical outcomes. (A–C) Tumor response rate, progression-free survival (PFS), and overall survival (OS) in 49 patients with chemo-naive *EGFR*-positive NSCLC and responder or non-responder PDCs. (**D–F**) Tumor response rate, progression-free survival (PFS), and overall survival (OS) in 10 patients with chemo-naive *ALK*-positive NSCLC and responder or non-responder PDCs.

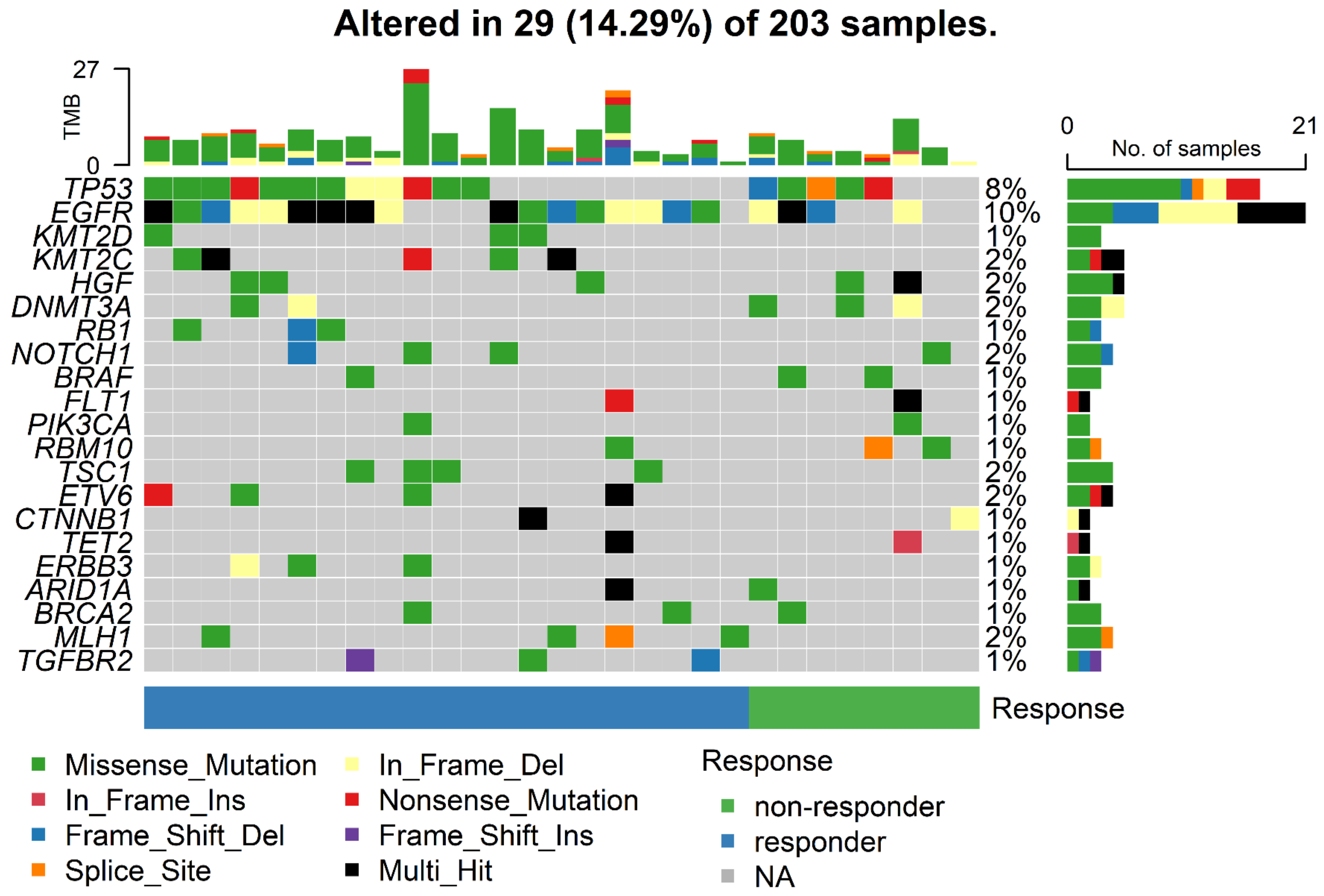

Figure S3. Heatmap comparing genetic variant profiles between PDC responders (n = 21) and PDC non-responders (n = 8).

(A)

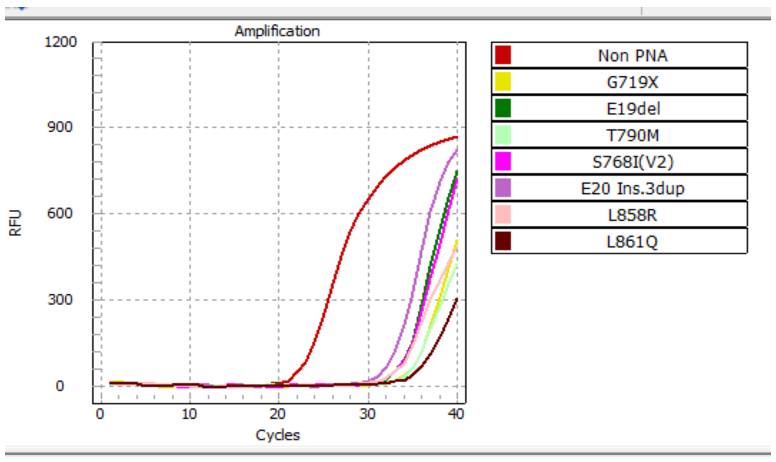

(B)

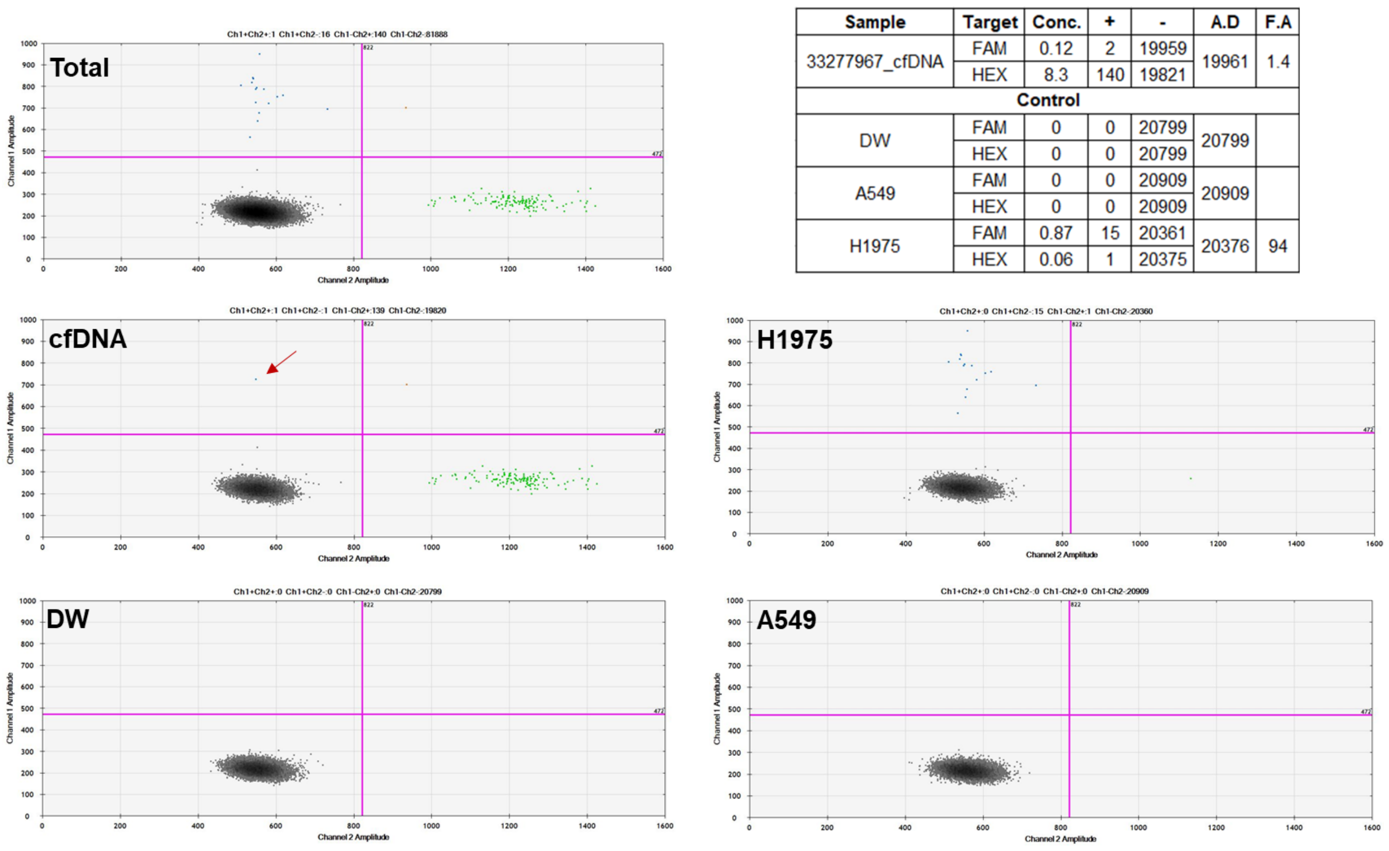

Sample	Target	Conc.	+	-	A.D	F.A
33277967_cfDNA	FAM	0.12	2	19959	19961	1.4
	HEX	8.3	140	19821		
Control						
DW	FAM	0	0	20799	20799	
	HEX	0	0	20799		
A549	FAM	0	0	20909	20909	
	HEX	0	0	20909		
H1975	FAM	0.87	15	20361	20376	94
	HEX	0.06	1	20375		

Figure S4. Results of *EGFR* mutation test in tumor NCCLu-027. **(A)** Cobas® mutation assay using tumor tissue DNA. **(B)** Droplet digital polymerase chain reaction test using plasma ctDNA. Red arrows indicate dots specific to the *EGFR* L858R mutation.

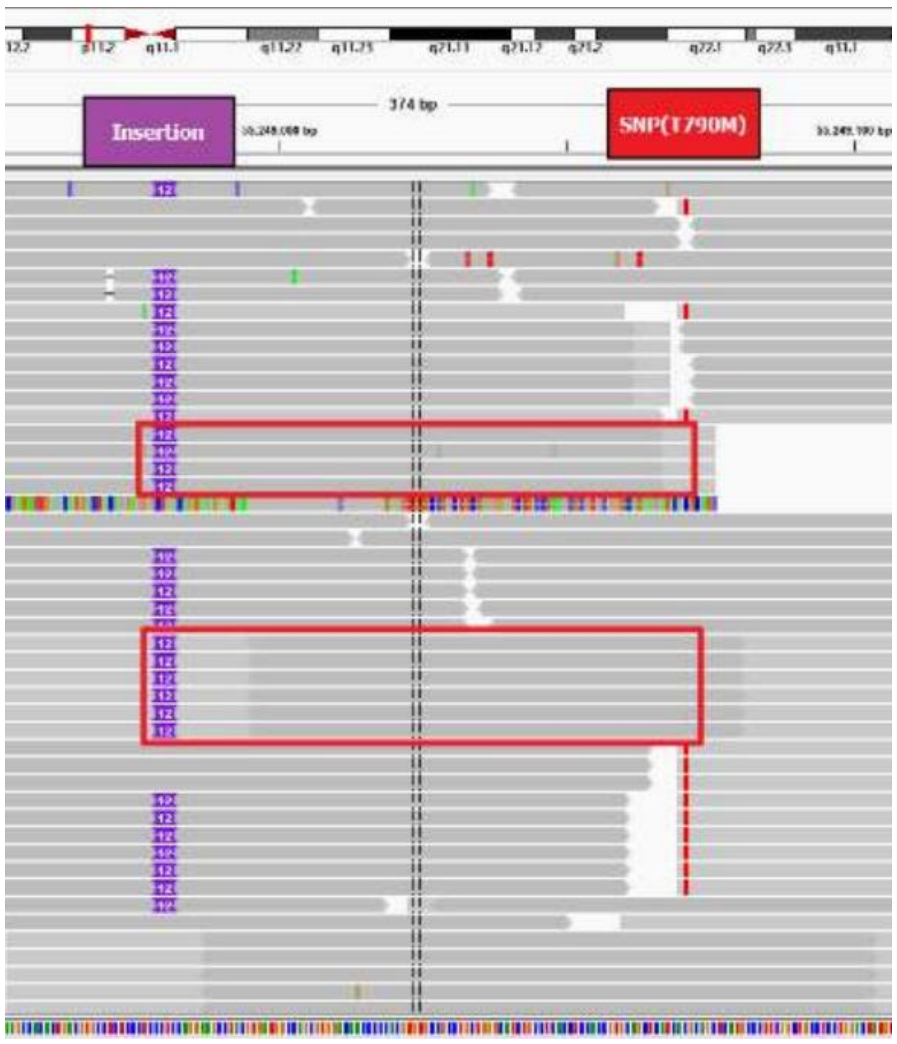

Figure S5. Targeted sequencing of tumor NCCLu-157 after it had developed erlotinib resistance. The secondary *EGFR* resistance mutation of exon 20 T790M present in *cis* with exon 20 A763_Y764insFQEA was identified.
