# Supplementary material for: Drug Response of Patient-Derived Lung Cancer Cells Predicts Clinical Outcomes of Targeted Therapy
Source: Cancers (Basel). 2024 Feb 14;16(4):778. doi: 10.3390/cancers16040778 (PMC10887363; doi:10.3390/cancers16040778)
Supplement: Supplementary file 1 [file cancers-16-00778-s001.zip › 5_PDC_Supple Tables_231227.pdf]

Table S1. Screening drug list

No.	Name	Target	Class	Screen
1	Adavosertib	Wee1	Cell cycle	226
2	Afatinib	EGFR	PTK (Protein Tyrosine Kinase)	391
3	AICAR	AMPK	Others	211
4	Alectinib	ALK	Others	272
5	Alisertib	Aurora A	cell cycle	75
6	Alpelisib	PI3K	PI3K/AKT/mTOR	334
7	AT7519	CDK1, 2, 4, 6 and 9	Cell cycle	175
8	auranofin	5-lipoxygenase	Inflammation	72
9	AZD4547	FGFR1/2/3	Others	211
10	AZD7762	Chk1	Cell cycle	390
11	Barasertib	Aurora Kinase	Cell cycle	329
12	Berzosertib	ATR	Cell cycle	229
13	Bosutinib	Src	Others	73
14	Brigatinib	ALK, FLT3,IGF1R,EGFR(C797S/del19)	Angiogenesis	73
15	Cabozantinib	VEGFR2, c-Met, Ret, Kit, Flt-1/3/4, Tie2 and AXL	Angiogenesis	269
16	Camptothecin	Topoisomerase	DNA damage	362
17	Capivasertib	AKT	PI3K/AKT/mTOR	210
18	CB-839	glutaminase inhibitor	metabolism	75
19	Ceritinib	ALK	Others	381
20	CHIR-99021	GSK-3	PI3K/Akt/mTOR	75
21	Cisplatin	DNA synthesis	DNA damage	360
22	Crizotinib	c-Met, ALK	Others	386
23	Dasatinib	Src, Bcr-Abl, c-Kit	Angiogenesis	233
24	Dinaciclib	CDK1, 2, 5 and 9	Cell cycle	188
25	Etoposide	Topoisomerase	DNA damage	361
26	Everolimus	mTOR	PI3K/AKT/mTOR	264
27	Fluoxentine	5-HT receptor	Neuronal Signaling	72
28	Fostamatinib	Syk	Others	72
29	Galunisertib	TGFbR1 (ALK5)	Others	197
30	Gefitinib	EGFR	PTK (Protein Tyrosine Kinase)	396
31	Gemcitabine	Deoxycytidine analogue	DNA damage	365
32	Ibrutinib	BTK	PTK (Protein Tyrosine Kinase)	234
33	Ivosidenib	IDH1	Others	211
34	JQ1	BRD	Others	75
35	Larotrectinib	TRK	Others	234
36	Lorlatinib	ALK/ROS1	Others	275
37	Navitoclax	Bcl-2	Others	299
38	Nilotinib	Bcr-Abl	Others	73
39	NOV1402	PARP/TNK	Others	72
40	Olaparib	PARP	Others	230
41	Omipalisib	PI3K	PI3K/Akt/mTOR	73
42	Osimertinib	EGFR	PTK (Protein Tyrosine Kinase)	229
43	Paclitaxel	Microtubule Associated	DNA damage	354
44	Palbociclib	CDK4/6	cell cycle	229
45	Pazopanib	VEGFR1, VEGFR2, VEGFR3, PDGFR, FGFR, c-Kit and c-Fms	PTK (Protein Tyrosine Kinase)	264
46	PD0166285	Wee1, Chk1	Cell cycle	207
47	Pemetrexed	Thymidylate synthase, DHFR	DNA damage	363
48	PF-562271	FAK	Others	209
49	Poziotinib	Pan_HER, HER1/2/4	PTK (Protein Tyrosine Kinase)	211
50	Pyrimethamine	DHFR, STAT3	Others	75
51	Repotrectinib	ALK/ROS1/TRK/SRC	Others	233
52	Rigosertib	PLK1	cell cycle	75
53	Ruxolitinib	JAK1/2	Others	230
54	Saracatinib	Src, Bcr-Abl	Angiogenesis	309
55	Selumetinib	MEK	MAPK	333
56	Sitravatinib	RTKs(RET, VEGFR, PDGFR, KIT), TRKs, DDR2, MET, AXL, cKit	PTK (Protein Tyrosine Kinase)	194
57	Tanespimycin	HSP90	Others	198
58	THZ1	CDK7	cell cycle	75
59	Torin 2	mTOR	PI3K/Akt/mTOR	75
60	Trametinib	MEK	MAPK	234
61	Vemurafenib	Raf	MAPK	234
62	Vismodegib	Hedgehog	Others	197
63	Vorinostat	HDAC	Others	197
64	XAV-939	Wnt/TNKS/beta-catenin	Others	225

Table S2. The origin of patient-derived cells

Specimen		No. of sampling	No. of successful drug screening test	Median time from sampling to report
Fluid	Pleural effusion	404	343 (84.9%)	15.3 days
	Pericardial effusion	16	15 (93.8%)	10.8 days
	Ascites	6	5 (83.3%)	13.9 days
	Cerebrospinal fluid	3	0 (0.0%)	-
	Urine	1	1 (100.0%)	16.0 days
Tissue	Aspirates and biopsies	27	11 (40.7%)	22.0 days
	Resected sample	30	21 (70.0%)	19.2 days
Total		487	397 (81.5%)	15.5 days

Table S3. Clinical characteristics of treatment-naive PDCs with *EGFR* mutation or *ALK* fusion

**Characteristics**		**Total No. (%)**
Total		59
Age	< 65 years	31 (52.5)
	≥ 65 years	28 (47.5)
Gender	Male	23 (39.0)
	Female	36 (61.0)
Histology	Adenocarcinoma	56 (94.9)
	Non-adenocarcinoma	3 (5.1)
Sample	Effusion	55 (93.2)
	Tissue	4 (6.8)
Oncogene	*EGFR* mutation	49 (83.1)
	ALK fusion	10 (16.9)
Drug	EGFR-targeted drug	
	Erlotinib	14 (28.6)
	Gefitinib	6 (12.2)
	Afatinib	2 (4.1)
	Dacomitinib	1 (2.0)
	Osimertinib	26 (53.1)
	ALK-targeting drug	
	Crizotinib	5 (50.0)
	Alectinib	3 (30.0)
	Ceritinib	1 (10.0)
	Brigatinib	1 (10.0)

Table S4. The classification of PDC drug sensitivity

Relative PDC drug response		Classification
The rank of AUC ≤ 25%	Highly sensitive	PDC responder
25% < The rank of AUC ≤ 50%	Moderate sensitive	
50% < The rank of AUC ≤ 75%	Moderate resistant	PDC non-responder
75% > The rank of AUC	Highly relative	

Table S5. Multivariate analysis of overall survival for EGFR-or ALK-tyrosine kinase inhibitors

**Characteristics**		**No.**	**Univariate analysis**		**Multivariate analysis**	
			**HR (95% CI)**	***P*†**	**HR (95% CI)**	***P*†**
Age	≥ 65 vs. <65 years	28 vs. 31	1.12 (0.56-2.25)	*0.747*		
Gender	Male vs. Female	23 vs. 36	1.65 (0.79-3.44)	*0.179*		
ECOG PS	0-1 vs. 2-3	41 vs. 18	0.27 (0.13-0.58)	*<0.001*	0.32 (0.15-0.68)	*0.003*
Smoking	Never vs. Ever	30 vs. 19	0.74 (0.36-1.51)	*0.405*		
Histology	ADC vs. non-ADC	56 vs. 3	0.65 (0.15-2.74)	*0.558*		
Operation	No vs. Yes	42 vs. 12	2.55 (1.05-6.21)	*0.040*	2.00 (0.76-5.22)	*0.160*
BM metastasis	No vs. Yes	41 vs. 18	0.39 (0.19-0.79)	*0.009*	0.67 (0.30-1.50)	*0.334*
TP53	WT vs. MT	14 vs. 11	0.76 (0.29-1.98)	*0.571*		
PDC response	Yes vs. No	40 vs. 19	0.50 (0.24-1.04)	*0.065*	0.58 (0.27-1.26)	*0.171*

Notes: †tested with Cox proportional hazards model. Abbreviations: HR, hazard ratio; CI, confidence interval; ECOG PS, ECOG performance status; ADC, adenocarcinoma; BM, brain metastasis; WT, wild type; MT, mutant type; TKI, tyrosine kinase inhibitor; PDC, patient-derived cell.

Table S6. Clinical, pathologic, and genetic characteristics of patients with *EGFR*-or *ALK*- positive NSCLC who had did not respond to EGFR or ALK-TKIs

	Patient No.	Histology	Companion diagnostics	Tissue/plasma targeted sequencing	PDC targeted sequencing	Targeted drug	Tumor response to targeted drug	Subsequent treatment	Possible cause of no response
1	NCCLu -034	LUAD	ALK-FISH: positive ALK-IHC: 1+	No variant	—	Crizotinib	PD	Alimta (PD)	False positivity
2	NCCLu -089	LUAD	ALK-IHC: 2+	EML4-ALK TP53 R175H, JAK2 V617F	TP53 R175H, HGF P8Q, DNMT3A R676W, PALB2 V1103M	Crizotinib	Overall PD: primary tumor (PR), pleural effusion/bone (PD)	Alimta+cisplatin (PD)	Tumor heterogeneity
3	NCCLu -131	LUAD	ALK-FISH: positive	BRAF V600E, TP53 W53*	BRAF V600E, P53 W53*, RBM10 splice site	Crizotinib	PD	Dabrafenib+ Trametinib (SD)	Tumor heterogeneity
4	NCCLu -237	LLC	ALK-IHC (D5F3): positive	EML4-ALK	No variant	Alectinib	PD	Alimta (PD)	Histology- neuroendocrine
5	NCCLu -099	LUAD	Real-time PCR EGFR-Cobas: EGFR 19del	EGFR 19del, PTEN C105fs8, TP53 G245fs*2	EGFR 19del, TP53G245fs, ARID1A T1345A, DNMT3A S638C, PTEN C105fs	Gefitinib	SD PFS: 2.9 mo	Osimertinib: PD, new lesion (liver)	Concurrent resistant mutation; PTEN loss
6	NCCLu-263	LUAD	Real-time PCR EGFR-Cobas: EGFR 19del	EGFR 19del, PIK3CA M1004I	EGFR 19del, PIK3CA M1004I, HGF E654Q, WHSC1L1 R780H, TET2 1421 insN, DNMT3A Y536del, SNCAIP Q118E, CSF1R G933S, PARP3 EAL234del, FLT1 S755Y, FLT1 E201D, MSH3 C822Y	Erlotinib	SD PFS; 3.1 mo	Alimta+cisplatin (PD)	Concurrent resistant mutation – PIK3CA
7	NCCLu -320	LUAD	Real-time PCR EGFR-Cobas: EGFR L858R	EGFR L858R, PIK3CA K111E	—	Osimertinib	PD	Alimta+cisplatin (PD)	Concurrent resistant mutation; PIK3CA

**8**	**NCCLu-253**	**LUSC**	**Real-time PCR EGFR-Cobas: EGFR 19del**	**—**	**—**	**Erlotinib**	**PD**	**Radiotherapy**	**Histology -squamous cell carcinoma**
**9**	**NCCLu -446**	**LUAD**	**Real-time PCR EGFR-Cobas: EGFR L858R**	**EGFR L858R, ACVR1B splice site 91+1G>T, CBL C384Y, CBL C404Y, DNMT3A splice site 1851+1G>A, TP53 E171G**	**EGFR L858R, TP53 E171G, BRCA2 K1533N, SMARCA4 K1349***	**Afatinib**	**PD**	**Atezolizumab+ bevacizumab+ taxol+carboplatin (PR)**	**Concurrent resistant mutation-ACVR1B loss**
**10**	**NCCLu -489**	**LUAD**	**Real-time PCR EGFR-Cobas: EGFR 19del/T790M**	**—**	**—**	**Osimertinib**	**SD PFS; 3.5 mo**	**none**	**Unknown**

Abbreviation: LUAD, lung adenocarcinoma; LUSC, lung squamous cell carcinoma; LCC, large cell carcinoma

Table S7. Clinical, pathologic, and genetic characteristics of patients who unexpectedly had response to *EGFR*-TKIs

	Patient No.	Histology	Companion diagnostics	Tissue/plasma a targeted sequencing	PDC targeted sequencing	Previous EGFR-TKI	Targeted drug	Tumor response to targeted drug	Categories
1	NCCLu-027	ADC	Real-time PCR EGFR-Cobas: EGFR wild type	PIK3CA E81K	ERBB3 E1098Q	none	Erlotinib	PR (PFS 22 mo)	Low-frequency mutation
2	NCCLu-157	ADC	Real-time PCR EGFR-Cobas: EGFR exon 20 insertion	EGFR A763_Y764insFQEA, TP53 T155N	EGFR A763_Y764insFQEA	none	Erlotinib	PR (PFS 8 mo)	Uncommon EGFR mutation
3	NCCLu-045	ADC	Real-time PCR EGFR-Cobas: EGFR L858R	EGFR L858R	EGFR L858R, EGFR I744M	Erlotinib, Olmutinib	Erlotinib	PR (PFS 11 mo)	Rechallenge with EGFR-TKI (TKI-free interval; 23 Mo)
4	NCCLu-254	ADC	Real-time PCR EGFR-Cobas: EGFR 19del	EGFR 19del, MET amp, CDKNA2 loss, NFE2L2, TP53 E271Q	EGFR 19del, ARID1A, NFE2L2	Erlotinib	Erlotinib	PR (PFS 2 mo, stop due to pneumonitis)	Rechallenge with EGFR-TKI (TKI-free interval; 7 Mo)
5	NCCLu-256	ADC	Real-time PCR EGFR-Cobas: EGFR 19del	—	—	Erlotinib	Osimertinib	PR (PFS 29 mo)	Rechallenge with EGFR-TKI (TKI-free interval; 10 Mo)
6	NCCLu-406	ADC	Real-time PCR EGFR-Cobas: EGFRL858R	EGFR L858R, PIK3CA E726K, GNAS R258L, BRCA1 E686*	—	Erlotinib	Osimertinib	PR (PFS 6 mo)	Rechallenge with EGFR-TKI (TKI-free interval; 9 Mo)
7	NCCLu-470	ADC	Real-time PCR EGFR-Cobas: EGFRL858R	EGFR L858R,	EGFR L858R, BRIP1, CDKN1A	Erlotinib	Erlotinib	PR (PFS 4 mo)	Rechallenge with EGFR-TKI (TKI-free interval; 11 Mo)

Abbreviation: ADC, adenocarcinoma
